# Supplementary material for: Native N-glycome profiling of single cells and ng-level blood isolates using label-free capillary electrophoresis-mass spectrometry
Source: Nat Commun. 2024 May 8;15:3847. doi: 10.1038/s41467-024-47772-w (PMC11079027; doi:10.1038/s41467-024-47772-w)
Supplement: Supplementary file 1 — Supplementary Information [file 41467_2024_47772_MOESM1_ESM.pdf]

Supplementary Materials for

Native N-glycome profiling of single cells and ng-level blood isolates using label-free capillary electrophoresis-mass spectrometry

Anne-Lise Marie, Yunfan Gao, and Alexander R. Ivanov*

* Corresponding author. E-mail: a.ivanov@northeastern.edu
Phone: +1 617 373 6549

Supplementary Figures 1-5

Supplementary Notes 1-4

Supplementary Methods

Supplementary References

Supplementary Note 1

IgM was selected to develop and optimize the label-free CE-MS method, which was thereafter applied to the analysis of other types of blood-derived isolates (IgG, total plasma, and total EV isolates), and small populations of cells and single cells. The injected amounts of biological samples were optimized depending on the concentration of the specific analyte type and the glycosylation level in the corresponding biological sample. For IgM and IgG, the lowest injected amounts (0.1 ng and 0.5 ng, respectively), corresponding to the total of ~10 pg of N-glycans for each protein, were equivalent to the amounts of IgM and IgG isolated from ~60 pL and ~50 pL of human serum, respectively (according to our estimations). These lowest injected amounts were selected for a comparative analysis with the injection of ~50 pL of total human plasma, and in order to release amounts of glycans corresponding, in theory, to the glycan content of one single mammalian cell (i.e., ~10-50 pg of glycans/single cell, on average [1]). For the total EV isolate, the highest injected amounts (equivalent to the EV content of ~150 nL of plasma) and the lowest injected amounts (equivalent to the EV content of ~3 nL of plasma) were selected based on the acquired MS signal intensity levels. Using these selected high and low amounts of EV isolate, the MS signal intensity levels of detected glycans in CE-MS analysis of the EV isolate corresponded to the signal levels recorded in CE-MS analysis of ~50 pL and ~5 pL of total plasma, respectively.

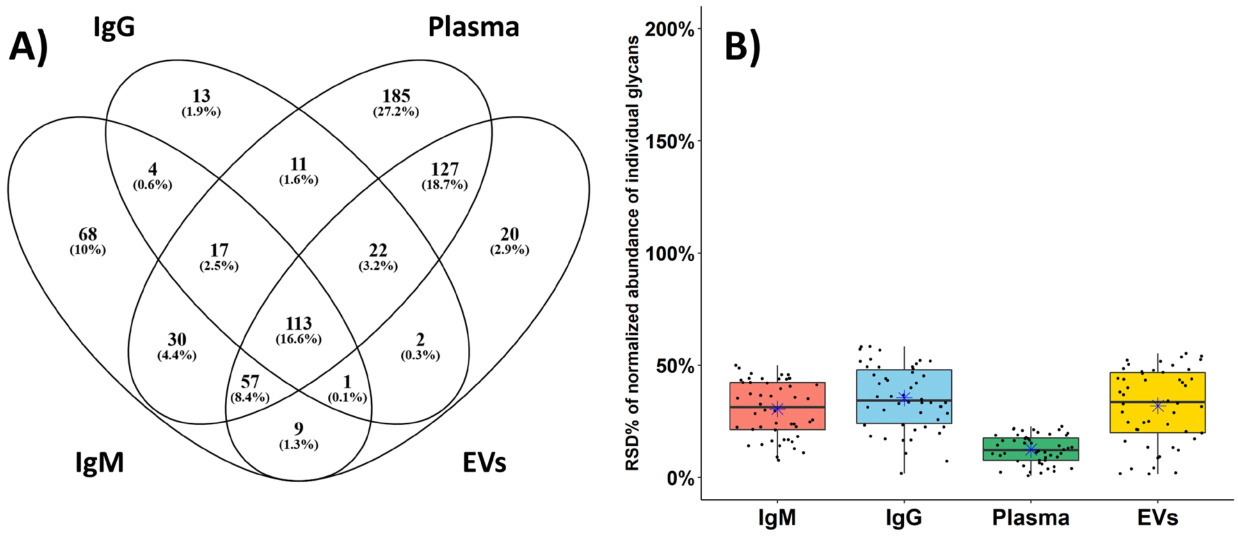

Supplementary Figure 1: Label-free CE-MS-based profiling of N-glycans released from human blood isolates (IgM, IgG, total plasma, and EVs). A) Venn diagram showing the overlap of N-glycans detected in the four analyzed blood-derived samples. B) Box plots showing the relative standard deviations (RSD) of glycan abundances measured in CE-MS analyses of the blood isolates, based on peak intensities (n=3 technical replicates, box plots display the median, first and third quartiles, and the whisker ends label minimum and maximum values, black dots correspond to individual data points, blue asterisks correspond to mean RSD values).

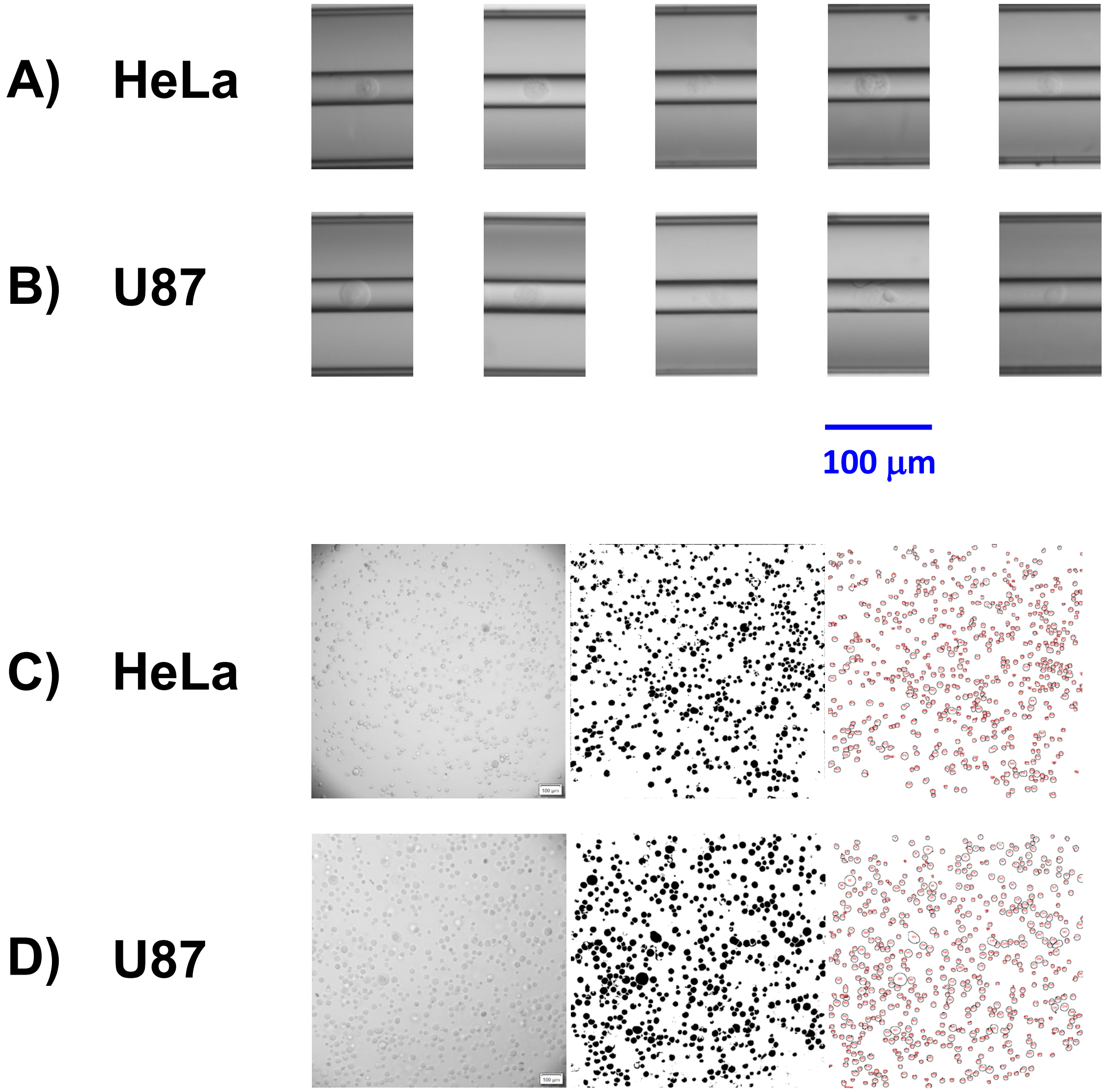

Supplementary Figure 2: Visualization of cell loading and cell morphology using our developed CE-MS-based workflow. Microscopic images (bright field) of the five single HeLa cells (A) and the five single U87 cells (B) selected and loaded in the CE capillary for subsequent in-capillary sample processing and CE-MS analysis. Microscopic images (bright field) of mammalian HeLa (C) and U87 (D) bulk cells and image processing to determine the cell morphology and size distribution.

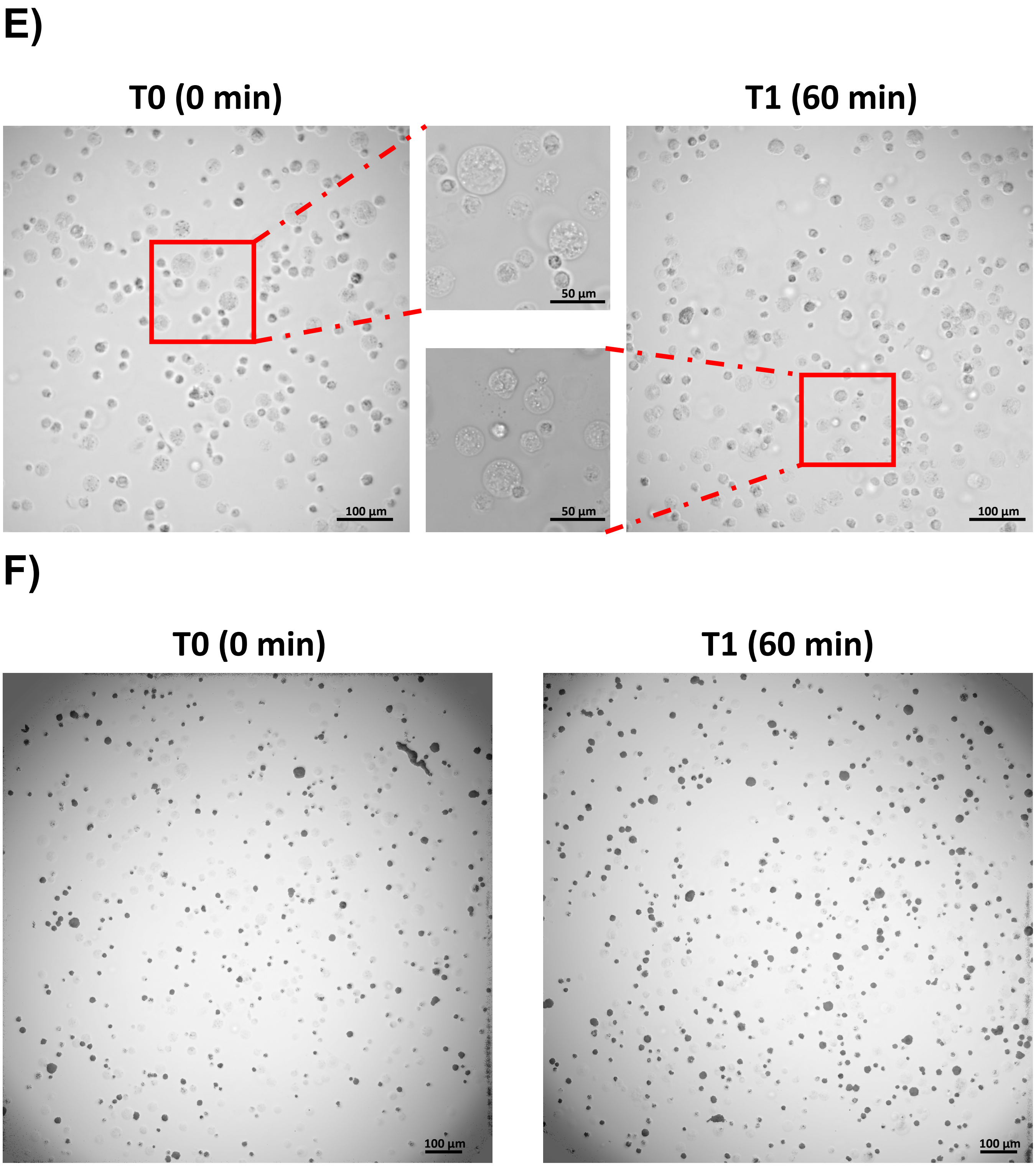

Supplementary Figure 2: Bright field images (E) and overlay of bright field and fluorescent images (F) of HeLa cells stained with LIVE/DEAD™ fixable green dye and incubated with PNGase F in 1 mM ammonium acetate pH 6.7 buffer. The microscopic images were recorded at two different time points during incubation with PNGase F: T0 (0 min) and T1 (60 min). The

fluorescence signal was converted to black and white for improved visualization of the cell viability.

Supplementary Note 2

Due to the relatively high amounts of salts or salt-associated molecular species in the analyzed biological samples, we assumed that the ion streaks detected in the ion density maps acquired in the CE-MS analyses of single, five, and ~ten cells corresponded to narrow bands of salts or other low molecular mass species contained in the sample matrices. The CE-MS analyses of single cells result in additional sources of variability, and, for such analyses, the matrix effect may be amplified due to the extremely low amounts of injected biological material. This hypothesis may explain a distinct CE-MS migration pattern for the analyzed single cells, in comparison to the CE-MS analyses of small populations of cells.

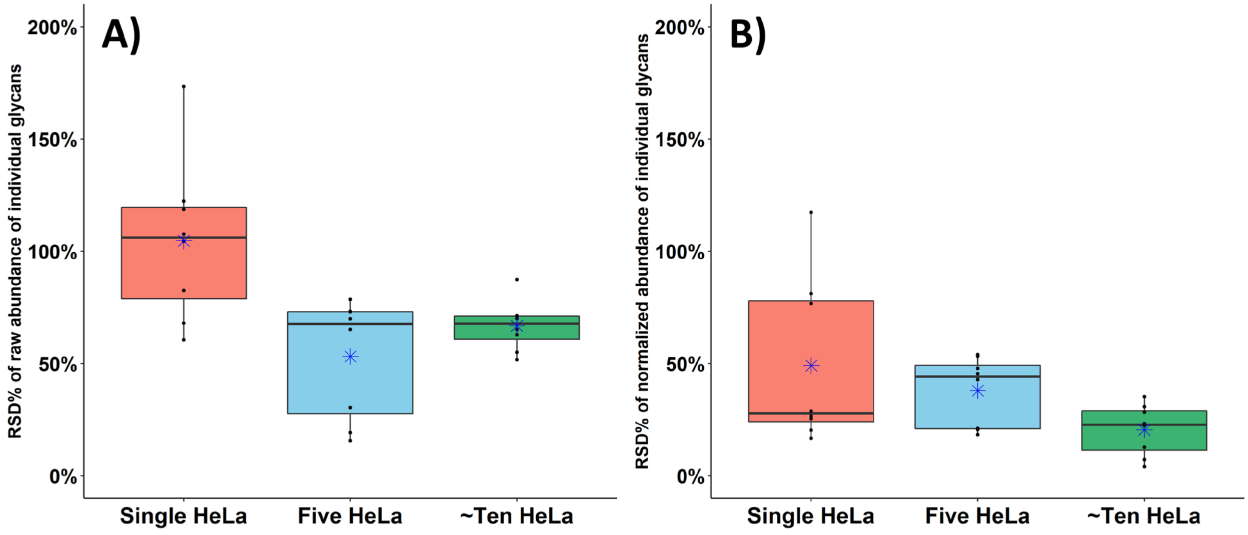

Supplementary Figure 3: Quantification of eight representative N-glycans detected in HeLa cells. Box plots showing (A) the RSD of raw abundance of individual N-glycans, and (B) the RSD of normalized abundance of individual N-glycans (i.e., each glycan abundance was normalized with respect to the summed abundances of the eight selected glycans) (n=5 technical replicates, box plots display the median, first and third quartiles, and the whisker ends label minimum and maximum values, black dots correspond to individual data points, blue asterisks correspond to mean RSD values).

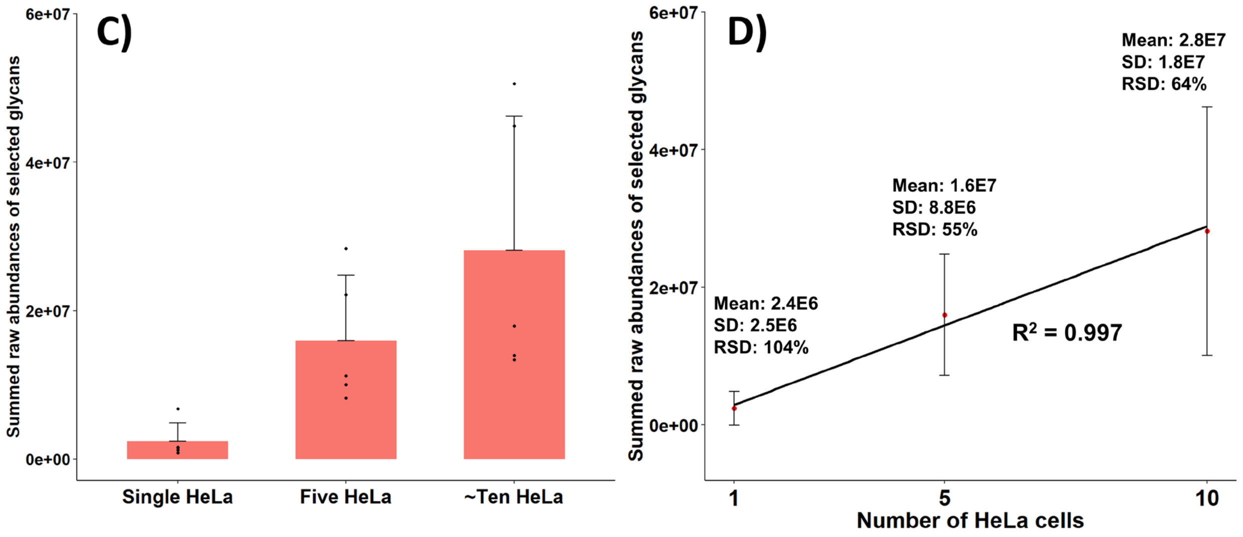

Supplementary Figure 3: Quantification of eight representative N-glycans detected in HeLa cells. C) Summed raw abundances of the eight selected N-glycans (n=5 technical replicates, data are presented as mean values ± SD, black dots correspond to individual data points). D) Linear fit of the summed raw abundances of the eight selected N-glycans against the number of HeLa cells injected in the CE capillary.

Supplementary Note 3

Due to cell-to-cell heterogeneity and cell size variations, high variations in the absolute abundances of glycans detected in single cells were observed. These trends were less pronounced in the CE-MS analyses of small populations of cells (i.e., 5-10 cells). Based on our experimental results (**Supplementary Figure 3CD**), the RSD of the summed raw abundances of eight representative N-glycans was 103% in the measurements of single cells, but was significantly lower, i.e., 55% and 64%, in the measurements of five and ~ten cells, respectively, based on peak areas (considering the variation in the number of cells injected in the CE-MS analysis of ~ten cells (see Methods section), the RSD in glycan abundances should be <64% for ~ten cells). Interestingly, as shown in **Supplementary Figure 3D**, a linear relationship was demonstrated between the injected cell numbers and the total cellular glycan amounts, based on peak area measurements. As shown in **Supplementary Figure 3AB**, the CE-MS analyses of small populations of cells (i.e., 5-10 cells) also resulted in decreased RSDs in the measurements of individual glycan abundances. The mean RSDs of the raw abundances of eight individual representative N-glycans were 105%, 53%, and 67%, in the measurements of single, five, and ~ten cells, respectively. These values were 49%, 38%, and 20%, respectively, for the normalized abundances of the same selected N-glycans.

Supplementary Note 4

The conducted proof-of-concept CE-MS^2 experiments, using the MS equipment we currently have access to in the lab, expectedly did not result in the structural characterization of all N-glycans that were detected and identified in the conducted CE-MS^1 analyses of the biological samples selected in this study. This may be explained by 1- the stochastic mode of data dependent acquisition of tandem mass spectra; 2- the low abundances of precursor ions of some glycans (in such cases, the acquired MS^2 spectra were insufficiently informative for reliable and accurate structural characterization); and 3- the limitations of the readily available glycan-dedicated software, glycan databases, and spectral libraries currently available for glycan identification.

A)

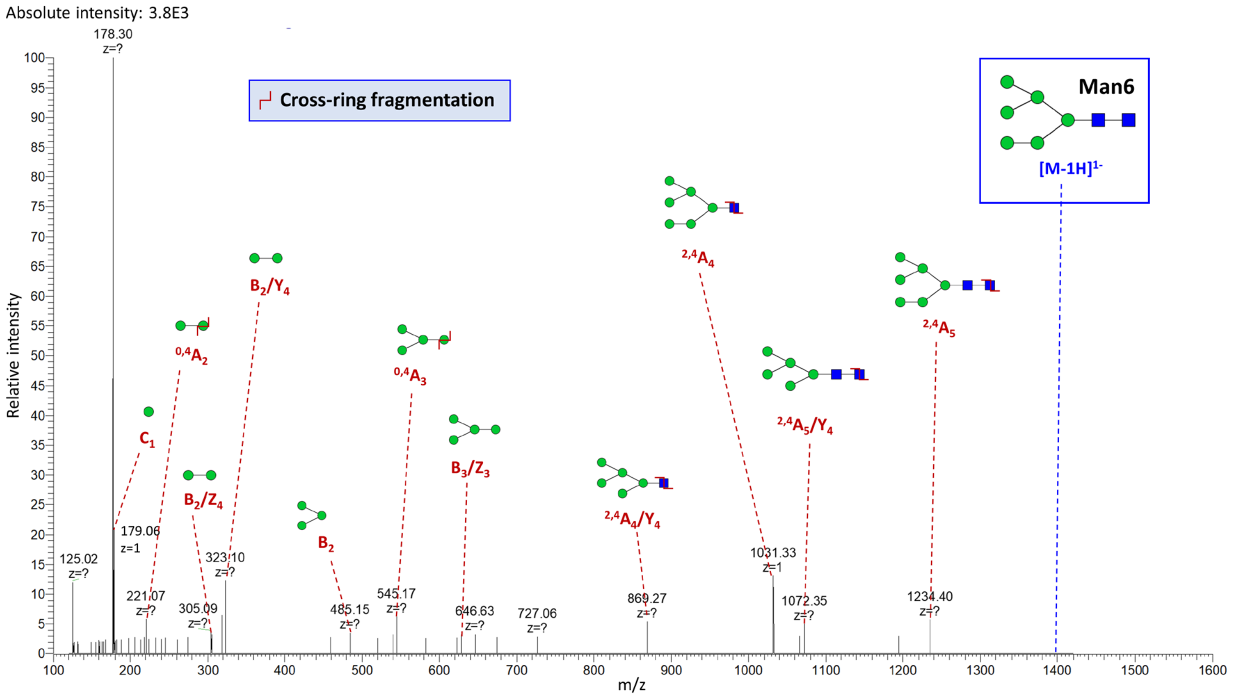

B)

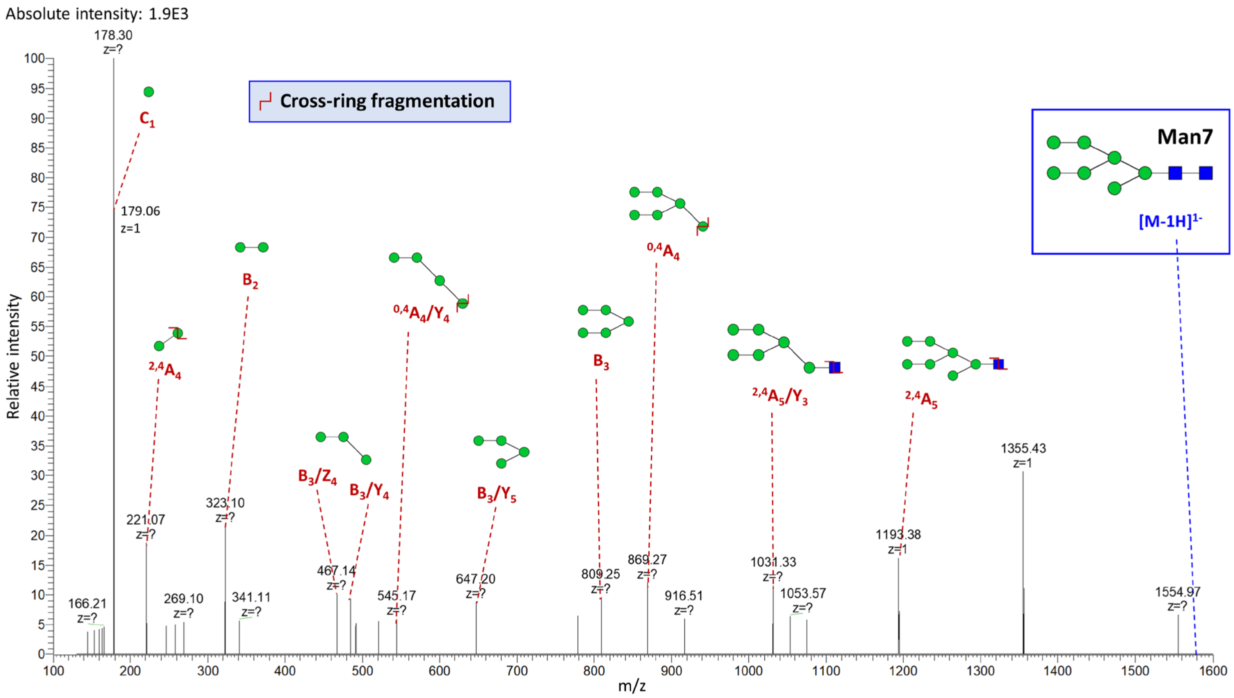

C)

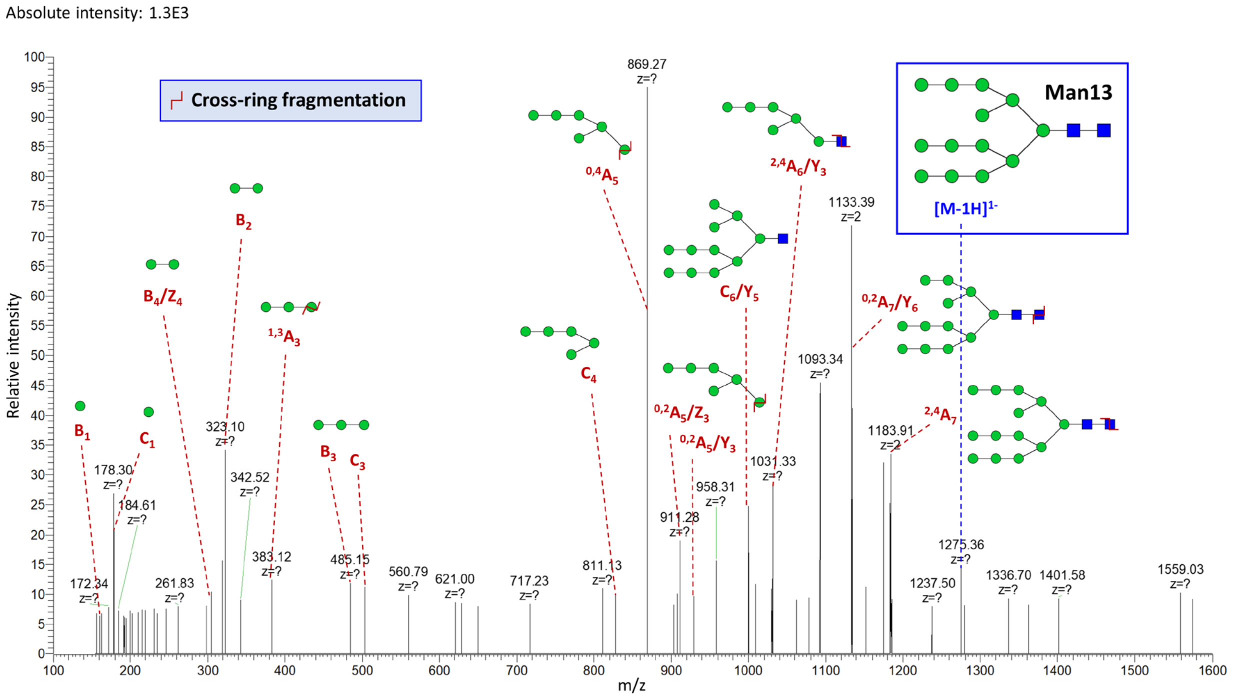

Supplementary Figure 4: Characteristic CE-MS^2 spectra of high mannose-type N-glycans detected in HeLa cells. The MS^2-based structural characterization of **Man6** (A) and **Man7** (B) was performed by selecting the $[M\text{-}1H]^{1-}$ molecular ions at *m/z* 1,395.47 and 1,579.53, respectively. The MS^2-based structural characterization of **Man13** (C) was performed by selecting the $[M\text{-}2H]^{2-}$ molecular ion at *m/z* 1,275.42.

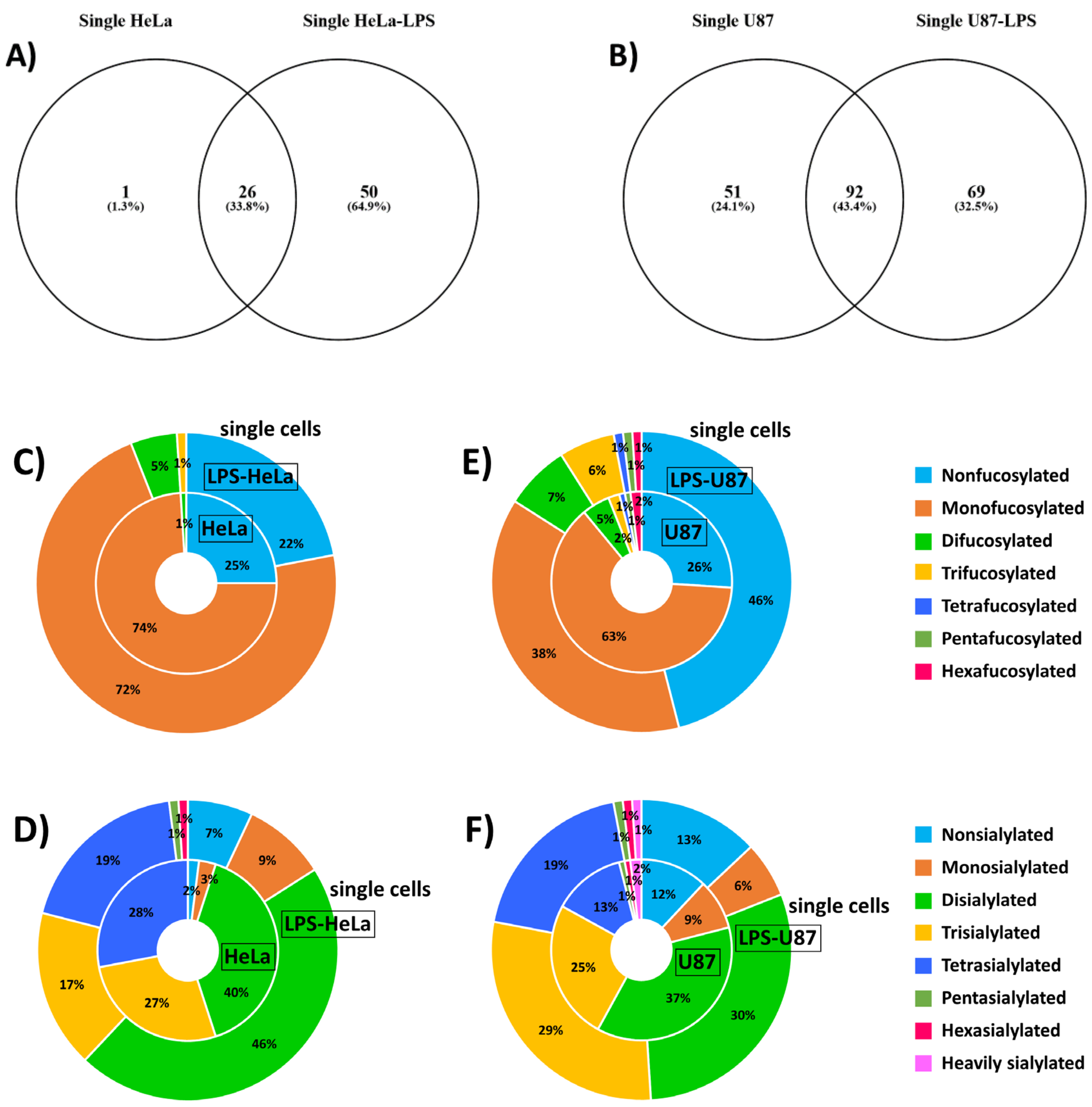

Supplementary Figure 5: Qualitative and quantitative comparison of single-cell N-glycomes before and after the stimulation of the cells with LPS. Venn diagrams illustrating the overlap of N-glycans identified in **single HeLa cells** before and after LPS treatment (A), and identified in **single U87 cells** before and after LPS treatment (B), based on five repetitive analyses for each cell line. Fractional abundances of fucosylated N-glycans detected in single HeLa (C) and single U87 (E) cells before and after LPS treatment. Fractional abundances of sialylated N-glycans detected in single HeLa (D) and single U87 (F) cells before and after LPS treatment.

Supplementary Methods

Determination of the hydrodynamic flow rate for sample injection. The volumes of the liquid solutions (blood isolates, cell culture medium, water, BGE, etc.) injected in the CE capillary by hydrodynamic pressure, using a CESI 8000™ instrument, were estimated based on the Hagen-Poiseuille equation (eq. 1). For the offline loading of one or five mammalian cells into the CESI OptiMS™ cartridge, a hydrodynamic flow was generated by manually lowering by ~45 cm the electrospray emitter tip of the capillary (i.e., the separation line outlet). The estimation of the theoretical hydrodynamic flow rate was based on the Hagen-Poiseuille's law (eq. 1) and eq. 2.

$$Q = \frac{\Delta P \cdot \pi \cdot d^4 \cdot t}{128 \cdot \eta \cdot L} \qquad \text{(eq. 1)}$$

Where Q represents the volumetric flow rate through the CE capillary; ΔP is the pressure difference between the inlet and outlet ends of the CE capillary (ΔP is equal to 4.3 x 10^3 Pa, based on eq. 2); d is the diameter of the capillary (i.e., 3 x 10^{-5} m); L is the length of the capillary (i.e., 9.0 x 10^{-1} m); and η is the viscosity of the BGE (~1.3 x 10^{-3} Pa.s).

$$\Delta P = \rho g h \qquad \text{(eq. 2)}$$

Where ρ is the density of the BGE (i.e., 9.86 x 10^2 kg/m^3); g is the gravity of earth (i.e., 9.8 m/s^2); and h is the height difference of the BGE (i.e., 4.5 x 10^{-1} m).

Based on equations 1 and 2, the theoretical hydrodynamic flow rate was ~7.4 x 10^{-14} m^3/s (i.e., ~74 pL/s).

The hydrodynamic flow rate generated by manually lowering the emitter tip by ~45 cm was also determined experimentally. For this, microscopy-based visualization was used to determine the laminar flow velocity, based on the assessment of the time required for an air plug to flow from point A to point B through a capillary filled with the BGE (i.e., 10 mM ammonium acetate pH 4.5 with 10% isopropanol). Briefly, one end of a bare fused silica capillary (30 µm ID x 150 µm OD x 90 cm length) was immobilized on a glass slide, placed under a microscope. To fill the capillary with the BGE, the other end of the capillary was inserted into a pressurized vial filled with the BGE. After the entire capillary was filled with the BGE, the capillary was removed from the pressurized vial and the free end of the capillary was lowered by ~45 cm to generate a height difference between both ends of the capillary. Driven by the height difference, the liquid in the capillary flowed towards the free end and generated an air plug at the immobilized end. The air plug was then trapped by adding a droplet of BGE to the immobilized end. Using the air plug as an indicator, the time required for the air plug to flow through a length of ~1.1 mm was recorded. The average flow velocity was calculated based on the exact length and flow time recorded in three replicate experiments. The determined laminar flow velocity was 6.7 ± 1.0 x 10^{-5} m/s (n=3), resulting in an estimated flow rate of 4.8 ± 0.7 x 10^{-14} m/s (i.e., 48 ± 7 pL/s), based on eq. 3.

$$Q = Av \qquad \text{(eq. 3)}$$

Where Q is the flow rate; A is the cross-sectional area of the capillary (i.e., 7.1 x 10^{-10} m^2); and v is the average laminar flow velocity.

Stacking strategy. In this study, a stacking strategy was used to increase the peak intensities and sharpen the detected peaks, for optimized detection and separation of the glycans, after their in-capillary release with PNGase F. To enable this strategy, we tried to highly decrease the concentrations of salts present in 1- the cell culture medium used to inject the mammalian cells inside the capillary, and 2- the commercial PNGase F solution. Therefore, the commercial PNGase F enzyme was diluted 7-fold in water, and the cells were resuspended in 1 mM ammonium acetate pH 6.7, immediately prior to their loading into the CE capillary. We noticed that this low molarity (i.e., 1 mM) of ammonium acetate buffer enabled the preservation of the cell integrity during the injection inside the capillary as well as during the deglycosylation step for N-glycan release. Nevertheless, to decrease the salt concentrations in the sample zone further, a short water plug was injected after the cell loading in the capillary, before the injection of a second PNGase F plug. These conditions helped the formation of a low conductivity zone after the mixing of the sample plug, water plug, and PNGase F plugs, following the application of two voltage pulses. After the switching of the CE voltage, the glycans released in a low conductivity zone migrate fast and are stacked as a sharp band at the boundaries between the sample and BGE zones. The short water plug injected after the sample injection was also performed to clean the CE capillary inlet before the second PNGase F plug injection in order to avoid potential cross-contaminations between the sample and endoglycosidase solutions.

Preparation of blood plasma isolate. The total plasma isolate was prepared from platelet-free anticoagulated with ethylenediaminetetraacetic acid (EDTA) pooled total human blood (donated by healthy male donors of mixed races and ethnicities of 23-67 years old) using differential centrifugation, as adapted from previously published work [2]. Briefly, human blood was collected in EDTA-containing vacutainer tubes. Then, total blood was centrifuged at 500 x g for 10 min to obtain total plasma, followed by two plasma centrifugations: 1,000 x g for 10 min and 10,000 x g for 10 min at room temperature to remove cell debris. Plasma was centrifuged at room temperature to prevent activation of platelets. Prior to CE-MS analysis, 1 mL of total plasma isolate was centrifuged at 16,000 x g for 20 min at 4 ºC, and the supernatant was carefully pipetted to avoid collecting the lipid layer. No protein depletion or enrichment was performed for the plasma samples, except for a partial removal of lipids.

CE-MS[1] data processing with GlycReSoft. According to the software developers' description [3], GlycReSoft uses a composite summarization score, which takes into account metrics including, inter alia, chromatographic peak shapes, ion charge state distributions, isotopic pattern consistency, adduct frequency, and time gap between single-stage MS observations (for missing peak and interference detection) to distinguish the observed features from MS background noise. For the assignment of glycan compositions to the chromatographic features, GlycReSoft utilizes the biosynthetic network relationship among glycan compositions and their neighboring subtypes. A Laplacian regularization algorithm is applied to combine the observed score and the glycan network graph topology to generate a smoothed score for the final glycan composition assignment.

Supplementary References

1 Misevic, G. Single-cell omics analyses with single molecular detection: challenges and perspectives. *J Biomed Res* **35**, 264-276, doi:10.7555/JBR.35.20210026 (2021).

2 Danielson, K. M. *et al.* Diurnal Variations of Circulating Extracellular Vesicles Measured by Nano Flow Cytometry. *PLoS One* **11**, e0144678, doi:10.1371/journal.pone.0144678 (2016).

3 Klein, J., Carvalho, L. & Zaia, J. Application of network smoothing to glycan LC-MS profiling. *Bioinformatics* **34**, 3511-3518, doi:10.1093/bioinformatics/bty397 (2018).
