# Supplementary material for: Native N-glycome profiling of single cells and ng-level blood isolates using label-free capillary electrophoresis-mass spectrometry
Source: Nat Commun. 2024 May 8;15:3847. doi: 10.1038/s41467-024-47772-w (PMC11079027; doi:10.1038/s41467-024-47772-w)
Supplement: Supplementary file 2 — Reporting Summary [file 41467_2024_47772_MOESM2_ESM.pdf]

Corresponding author(s): Alexander Ivanov
Last updated by author(s): 03/29/2024

Reporting Summary

Nature Portfolio wishes to improve the reproducibility of the work that we publish. This form provides structure for consistency and transparency in reporting. For further information on Nature Portfolio policies, see our Editorial Policies and the Editorial Policy Checklist.

Statistics

For all statistical analyses, confirm that the following items are present in the figure legend, table legend, main text, or Methods section.

n/a	Confirmed	
☐	☒	The exact sample size (*n*) for each experimental group/condition, given as a discrete number and unit of measurement
☐	☒	A statement on whether measurements were taken from distinct samples or whether the same sample was measured repeatedly
☐	☒	The statistical test(s) used AND whether they are one- or two-sided<br>*Only common tests should be described solely by name; describe more complex techniques in the Methods section.*
☒	☐	A description of all covariates tested
☒	☐	A description of any assumptions or corrections, such as tests of normality and adjustment for multiple comparisons
☐	☒	A full description of the statistical parameters including central tendency (e.g. means) or other basic estimates (e.g. regression coefficient) AND variation (e.g. standard deviation) or associated estimates of uncertainty (e.g. confidence intervals)
☐	☒	For null hypothesis testing, the test statistic (e.g. *F*, *t*, *r*) with confidence intervals, effect sizes, degrees of freedom and *P* value noted<br>*Give P values as exact values whenever suitable.*
☒	☐	For Bayesian analysis, information on the choice of priors and Markov chain Monte Carlo settings
☒	☐	For hierarchical and complex designs, identification of the appropriate level for tests and full reporting of outcomes
☒	☐	Estimates of effect sizes (e.g. Cohen's *d*, Pearson's *r*), indicating how they were calculated

Our web collection on statistics for biologists contains articles on many of the points above.

Software and code

Policy information about availability of computer code

Data collection	For data acquisition, Xcalibur (v. 3.1) software was used.
Data analysis	For data processing, Xcalibur (v. 3.1) software was used. CE-MS1 and CE-MS2 data were processed with GlycReSoft (v. 3.10) software (Boston University, Boston, MA, USA) and SimGlycan (v. 5.91) software (Premier Biosoft, Palo Alto, CA, USA), respectively. The bar charts with individual data points, mean values, and error bars were plotted using the R language and ggplot2 package in the rStudio development environment (2023.03.0+386 "Cherry Blossom" Release). The R language in the rStudio development environment was also used to perform statistical ANOVA and paired t-tests. The open-access tBtools-II (v1.120) software was employed to generate heatmap clustering, utilizing the Euclidean distance-based clustering method and the complete cluster approach. The PCA plots were created with the open-access version of ClustVis (https://biit.cs.ut.ee/clustvis) software. The average cell diameters of HeLa and U87 cells were measured using the open-access ImageJ (v1.53k) software.

For manuscripts utilizing custom algorithms or software that are central to the research but not yet described in published literature, software must be made available to editors and reviewers. We strongly encourage code deposition in a community repository (e.g. GitHub). See the Nature Portfolio guidelines for submitting code & software for further information.

Data

Policy information about availability of data

All manuscripts must include a data availability statement. This statement should provide the following information, where applicable:

- Accession codes, unique identifiers, or web links for publicly available datasets
- A description of any restrictions on data availability
- For clinical datasets or third party data, please ensure that the statement adheres to our policy

Data availability: The raw data generated in this study have been deposited in GlycoPOST (https://glycopost.glycosmos.org) under the accession numbers GPST000378 and GPST000380. Source data are provided with this paper.

Research involving human participants, their data, or biological material

Policy information about studies with human participants or human data. See also policy information about sex, gender (identity/presentation), and sexual orientation and race, ethnicity and racism.

Reporting on sex and gender	Male donors.
Reporting on race, ethnicity, or other socially relevant groupings	Self-declared healthy male volunteer donors of various races and ethnicities of the age 23 - 67 years old.
Population characteristics	Self-declared healthy male volunteer donors of the age 23 - 67 years old.
Recruitment	Twelve self-declared healthy male volunteer donors were recruited, and informed consent was obtained for each participant. A compensation of $10 was provided to the donors.
Ethics oversight	The research study involving human subjects was reviewed by the respective authorized Institutional Review Boards (IRB) with approvals IRB#2001P000S91 (BIDMC) and IRB#17-12-14 (NU).

Note that full information on the approval of the study protocol must also be provided in the manuscript.

Field-specific reporting

Please select the one below that is the best fit for your research. If you are not sure, read the appropriate sections before making your selection.

☒ Life sciences ☐ Behavioural & social sciences ☐ Ecological, evolutionary & environmental sciences

For a reference copy of the document with all sections, see nature.com/documents/nr-reporting-summary-flat.pdf

Life sciences study design

All studies must disclose on these points even when the disclosure is negative.

Sample size	The sample size was selected based on the number of replicate experiments required to reliably determine the reproducibility and standard deviation of the proof-of-concept method (i.e., at least three replicate experiments were conducted for each sample type).
Data exclusions	Infrequent failures in experiments were caused by hardware malfunction (mechanical capillary damage). Such failures led to unsuccessful measurements that were excluded from this study.
Replication	The run-to-run and day-to-day reproducibility was assessed using at least three (blood isolates) and five (mammalian cells) replicates. All replicate experiments were successful if no capillary damage occured. Infrequent failures in experiments were caused by hardware malfunction (mechanical capillary damage). Such failures led to unsuccessful measurements that were excluded from this study.
Randomization	N/A Different types of model samples (blood isolates) and mammalian cells were selected for evaluation of the developed approach. Since the comparative analysis of the selected samples was not the goal of this study, randomization was not deemed necessary in this study.
Blinding	N/A Different types of model samples (blood isolates) and mammalian cells were selected for evaluation of the developed approach. Since the comparative analysis of the selected samples was not the goal of this study, blinding was not deemed necessary in this study.

Reporting for specific materials, systems and methods

We require information from authors about some types of materials, experimental systems and methods used in many studies. Here, indicate whether each material, system or method listed is relevant to your study. If you are not sure if a list item applies to your research, read the appropriate section before selecting a response.

Materials & experimental systems

n/a	Involved in the study
☒	☐ Antibodies
☐	☒ Eukaryotic cell lines
☒	☐ Palaeontology and archaeology
☒	☐ Animals and other organisms
☒	☐ Clinical data
☒	☐ Dual use research of concern
☒	☐ Plants

Methods

n/a	Involved in the study
☒	☐ ChIP-seq
☒	☐ Flow cytometry
☒	☐ MRI-based neuroimaging

Eukaryotic cell lines

Policy information about cell lines and Sex and Gender in Research

Cell line source(s)	HeLa-S3 and U87-MG cell lines were from ATCC (Manassas, VA).
Authentication	None of the cell lines were authenticated since they were commercial samples supplied by ATCC.
Mycoplasma contamination	None of the cell lines were tested for mycoplasma contamination since they were commercial samples supplied by ATCC.
Commonly misidentified lines (See ICLAC register)	N/A - No commonly misidentified cell lines were used in the study.

Plants

Seed stocks	N/A
Novel plant genotypes	N/A
Authentication	N/A
