# Supplementary material for: Native N-glycome profiling of single cells and ng-level blood isolates using label-free capillary electrophoresis-mass spectrometry
Source: Nat Commun. 2024 May 8;15:3847. doi: 10.1038/s41467-024-47772-w (PMC11079027; doi:10.1038/s41467-024-47772-w)
Supplement: Supplementary file 3 — Description of Additional Supplementary Files [file 41467_2024_47772_MOESM3_ESM.pdf]

Description of Additional Supplementary Files

File name: **Supplementary Data 1**
Description: Average number and standard deviation (SD) of N-glycans detected in blood-derived isolates and mammalian cells.

File name: **Supplementary Data 2**
Description: Euclidean distance-based hierarchical clustering of quantitative glycomic profiles of IgM, IgG, total plasma, and total EV isolates, based on normalized glycan signal intensities. The order of the glycans listed in Supplementary Data 2 corresponds to the circular heatmap in Figure 2J.

File name: **Supplementary Data 3**
Description: N-glycan structures characterized in label-free CE-MS^2 analyses of HeLa cells.

File name: **Supplementary Data 4**
Description: Statistical tests (one-way ANOVA and two-sided paired t-tests) in CE-MS analyses of N-glycans derived from HeLa and U87 cells.

File name: **Supplementary Data 5**
Description: Euclidean distance-based hierarchical clustering of quantitative glycomic profiles of single HeLa and single U87 cells, based on normalized glycan signal intensities. The order of the glycans listed in Supplementary Data 5A and 5B corresponds to the linear heatmaps in Figure 6A and 6C, respectively.

File name: **Supplementary Data 6**
Description: N-glycan structures characterized in label-free CE-MS^2 analyses of U87 cells.
